# H- and m-channel overexpression promotes seizure-like events by impairing the ability of inhibitory neurons to process correlated inputs

**DOI:** 10.1371/journal.pcbi.1013199

**Published:** 2025-06-30

**Authors:** Scott Rich, Taufik A. Valiante, Jérémie Lefebvre

**Affiliations:** 1 Department of Physiology and Neurobiology, University of Connecticut, Storrs, Connecticut, United States of America; 2 Departments of Mathematics and Biomedical Engineering and Institute for Brain and Cognitive Sciences, University of Connecticut, Storrs, Connecticut, United States of America; 3 Division of Clinical and Computational Neuroscience, Krembil Brain Institute, Toronto, Ontario, Canada; 4 Institute of Biomaterials and Biomedical Engineering and Institute of Medical Science, University of Toronto, Toronto, Ontario, Canada; 5 Division of Neurosurgery, Department of Surgery, University of Toronto, Toronto, Ontario, Canada; 6 Electrical and Computer Engineering, University of Toronto, Toronto, Ontario, Canada; 7 Center for Advancing Neurotechnological Innovation to Application (CRANIA), University of Toronto, Toronto, Ontario, Canada; 8 Max Planck-University of Toronto Centre for Neural Science and Technology, University of Toronto, Toronto, Ontario, Canada; 9 Department of Biology, University of Ottawa, Ottawa, Ontario, Canada; 10 Department of Mathematics, University of Toronto, Toronto, Ontario, Canada; University of Pittsburgh, UNITED STATES OF AMERICA

## Abstract

Channelopathies affecting the hyperpolarization-activated cyclic nucleotide gated (HCN or h-) channel and the Kv7 voltage gated m-type potassium (m-) channel present a paradox in epilepsy research: despite experimental evidence that both over- and underexpression of these channels can be epileptogenic, channel overexpression does not appear to increase the excitatory-inhibitory (E-I) balance as caused by channel underexpression. We here derive a viable mechanism for ictogenesis driven by h- and m-channel overexpression from analysis of an *in silico* spiking neuronal microcircuit exhibiting spontaneous seizure-like events (SLEs). Such SLEs are dependent upon sufficiently strong gain in two adaptation terms phenomenologically modeling these channels’ effects: voltage homeostasis (h-current) and spike-frequency adaptation (m-current). Excessive gain of these adaptation terms translates high levels of input correlation into population-level deviations from baseline activity, promoting a sequence of network-level events that collectively provoke an SLE. Importantly, these changes do not cause increased excitability in isolated neurons, nor does this cascade require a change in the amplitude of external input to the circuit, suggesting an ictogenic pathway independent of classical changes to the E-I balance. The viability of this mechanism for SLE onset is strengthened by the host of experimentally-characterized features of seizure produced in this model reliant upon the presence of these adaptation terms, including the irregular initiation and termination of SLEs and time-varying peak frequency of oscillations during such events (i.e., chirps). Moreover, the cell-type dependent effects of changes in these adaptation terms, as delineated in our analyses, represent experimentally-testable predictions for future study of h- and m-channelopathies. These computational results provide vital new insights into the epileptogenic nature of h- and m-channel overexpression currently absent in the experimental literature.

## Author summary

How a vast array of insults to the healthy brain can all lead to the same pathological endpoint of seizure remains one of the most perplexing questions in epilepsy research. Historically, computational modeling has been successfully used to derive mechanisms relating many such insults to seizure onset. These pathways are often classified by how particular insults perturb the balance between excitation and inhibition in neuronal microcircuits; as an example, underexpression of both the h- and m-channels are thought to lead to seizure by promoting excessive excitation or insufficient inhibition. This intuition, though, is challenged by the seeming paradox that the *over*expression of both these ion channels is also observed in some epilepsies. Here, we address this lingering question using *in silico* approaches by first creating a model that produces seizure-like events dependent upon approximations of the effects of excessive h- and m-channel expression. Analyzing this model leads to a new hypothesis for how these pathologies promote seizure without contradicting previous findings on channel underexpression: such changes instead alter the network’s response to unusually correlated inputs. This mechanism recontextualizes our understanding of this relatively understudied cause of epilepsy, providing a host of experimentally testable predictions for future study.

## Introduction

Epilepsy, the most common serious neurological disorder in the world [1], is typified by the brain’s predisposition to repeated and unpredictable transitions into hyperactive oscillatory states called seizures [2]. Despite important advances, understanding the myriad etiologies underlying epileptogenesis and ictogenesis remains a formidable challenge [3–14]. Many of these are jointly classified as channelopathies, with the pathological impetus promoting seizure lying in mutations to genes encoding ion channels [15–18]. Two ion channels of particular interest are the hyperpolarization-activated cyclic nucleotide gated (HCN or h-) channel and the Kv7 voltage gated m-type potassium (m-) channel, which drive key homeostatic and adaptive processes in neurons [19–24] and have thus emerged as key regulators of neuronal excitability and function. H- and m-channelopathies have been implicated in epileptogenesis [10,25–33] and control of these channels’ activity has shown promise in seizure prevention [25,26,34].

However, the relationship between these channels and seizure appears surprisingly complex. For example, both over- [29–33] and under- [10,29,30,33] expression of the h-channel can be epileptogenic. While the effects of h-channel underexpression are intuitively understood through effects on a neuronal population’s excitatory-inhibitory (E-I) balance, mechanisms explaining the epileptogenic effects of h-channel overexpression are relatively understudied. Similarly, while decreased expression of the m-channel is more commonly associated with epilepsy [25–28] given the m-current’s role in driving spike-frequency adaptation [19,35,36], recent studies have identified epileptogenic gain of function mutations related to the m-channel [37]. As both these channels play a role in controlling neuronal excitability—the h-channel through voltage homeostasis and the m-channel through spike-frequency adaptation—it is possible that excessive excitability control caused by either channel’s overexpression might trigger seizure via similar pathways. Mechanistic explanations for these less intuitive epileptogenic effects of h- and m-channel overexpression could yield new therapeutic targets for the approximately one-third of epilepsy patients who do not respond to current pharmaceutical interventions [38,39].

*In silico* tools are ideally suited to decipher these relationships, as a host of computational studies [6,13,40–54] have described detailed dynamical and physiological mechanisms underlying epileptiform activity. Here, we build on these approaches and our previous work [6,53] to explore the role of h- and m-channel misexpression in seizure susceptibility. Specifically, we built a spiking E-I neuronal microcircuit accounting for activity driven by the h- and m-channels by endowing each neuron with a voltage homeostasis current and a spike-frequency adaptation term. The former is a computationally tractable phenomenological implementation of the h-current’s activation when the membrane voltage is hyperpolarized [20] and therefore referred to as h-adaptation, while the latter reflects the m-current’s key contribution to spike-frequency adaptation [19,35,36] and is therefore referred to as m-adaptation. Our previous work showed that this network architecture (without adaptation) would suddenly transition into a hyperactive oscillatory state via a saddle-node bifurcation [6,53,55] with increasing tonic external input. Here, we expose the new model to dynamic stimuli in the form of noisy inputs with varying correlation, emulating fluctuations arising naturally due to recurrent circuit connectivity [56], synchrony [57], and/or common afferent inputs including sensory stimuli [58–63]. Such inputs interrogate the network’s stability and, in turn, susceptibility to seizure-like events (SLEs).

By thoroughly exploring the parameter space defined by varying the strengths of these two adaptation terms, we illustrate that more frequent spontaneous [64,65] transitions into and out of SLEs arise with sufficiently strong h- and m-adaptation, with these sources of adaptation interacting in non-linear and cell-type dependent fashions. In fact, these adaptation terms were found to drive non-stationary oscillatory behavior commonly observed during seizure (i.e., chirps or “glissandi" [66,67]), in which neural activity traverses multiple dynamical regimes characterized by distinct spectral properties [68]. Mechanistically, we present computational evidence that strong h- and m-adaptation promote SLE susceptibility by corrupting the network’s response to correlated inputs, as the unique sensitivity of such systems’ to input correlation promotes more variable population activity during non-SLE periods. SLEs arise when the inhibition resulting from such fluctuations is translated into post-inhibitory hyperexcitability in the excitatory population via excessive h-adaptation, combined with inhibitory neurons’ inability to restrain this hyperexcitability due to excessive m-adaptation. We argue the effects of these adaptation terms on the microcircuit’s response to correlated input represents a unique mechanism for seizure onset driven by h- and m-channel overexpression distinct from effects on the E-I balance, which traditionally explain the ictogenic consequences of h- and m-channel underexpression. Our model further suggests that the ictogenic effects of h- and m-channel overexpression might be due in part to cell-type dependent effects: the epileptogenic effects of gain of function mutations promoting m-channel expression [37] can be explained by increased spike-frequency adaptation in inhibitory neurons overshadowing similar effects in excitatory neurons, and similarly the epileptogenic effects of h-channel overexpression [29–33] may arise when post-inhibitory excitability in excitatory cells overshadows effects on inhibitory neurons. These results provide new mechanistic insights into the epileptogenic effects of h- and m-channelopathies via a unique *in silico* seizure model reflecting the diverse etiologies of epilepsy.

## Materials and methods

### Model epileptogenic neuronal circuit

We present an epileptogenic spiking neuronal network model of recurrently connected excitatory and inhibitory neurons, inspired by numerous previous studies from our group [6,53] and others [13,40–52,54]. The model, which spontaneously transitions into SLEs, follows the general architecture of our previous work [6,53] with the important addition of of spike-frequency adaptation and voltage homeostasis.

The spiking response of those neurons obeys the non-homogeneous Poisson process

$$X_{j}\to Poisson(f(u_{j,x},h_{j}))$$ (1)

where $X_{j}=\sum_{k}\delta (t-t_{k})$ is a Poisson spike train (for spike times *t*_*k*_ of neuron *j*) with firing rate $f(u_{j,x},h_{j})$ defined by the sigmoidal response function

$$f(u_{j,x},h_{j})=\frac{1}{1+e^{-\beta (u_{j,x}-h_{j})}}$$ (2)

where *u*_*j*,*x*_ is the unitless membrane potential analogue for neuron *j*, and the index *x* = *e* when neuron *j* is an excitatory neuron while *x* = *i* when neuron *j* is inhibitory. In the equation above, *h*_*j*_ denotes a shift in this sigmoidal activation function for neuron *j* that sets its excitability, while β refers to its response gain, chosen to be uniform across the network. We note that terms akin to *h*_*j*_ are often referred to as the “rheobase" in the computational literature despite this term *not* defining the electrophysiological rheobase (i.e., the minimum input required for repetitive spiking). Consequently, the probability of neuron *j* firing at time *t* is dependent upon *u*_*j*,*x*_ and is equal to

$$\rho_{j}=1-e^{-f(u_{j,x},h_{j})dt}$$ (3)

for *dt* sufficiently small. Heterogeneity in the network is introduced via the *h*_*j*_, which are chosen by independently sampling a normal distribution with standard deviation $\sigma_{e,i}$ if the neuron is excitatory (*e*) or inhibitory (*i*). By default, $\sigma_{e,i}=0.01$, modeling very low heterogeneity.

The dynamical system defining the temporal evolution of the membrane potential analogue *u*_*j*,*x*_ is given by the following:

$$\alpha_{x}^{-1}\frac{du_{j,x}}{dt}=-\frac{1}{2}u_{j,x}+b^{h}v_{j,x}^{h}+b^{m}v_{j,x}^{m}+Syn_{ex}^{j}+Syn_{ix}^{j}+I_{x}\;+\sqrt{2D}\left(\sqrt{1-c}\chi_{u}^{j}+\sqrt{c}\chi_{c}\right)$$ (4)

$$(\alpha^{h}{)}^{-1}\frac{dv_{j,x}^{h}}{dt}=-v_{j,x}^{h}+\gamma^{h}\left(u_{j,x}-I_{x}\right)$$ (5)

$$(\alpha^{m}{)}^{-1}\frac{dv_{j,x}^{m}}{dt}=-v_{j,x}^{m}+\gamma^{m}X_{j}$$ (6)

for *x* = *e* when neuron *j* is excitatory, and *x* = *i* when neuron *j* is inhibitory. Note these equations describe the “default" scenario in which $\gamma^{h}$ and $\gamma^{m}$ are uniform across the excitatory and inhibitory populations, with only straightforward adjustments needed to instead reflect population-specific gains $\gamma_{e}^{h}$, $\gamma_{i}^{h}$, $\gamma_{e}^{m}$, and $\gamma_{i}^{m}$.

The variable $v_{j,x}^{h}$ is our modeled voltage-homeostasis term, that evolves proportional to the difference between the current value of *u*_*j*,*x*_ and the bias current *I*_*x*_. Considering that ${\text{b}}^{\text{h}}<0$ (see Table 1), this serves to excite the neuron when $u_{j,x}<I_{x}$ for a prolonged period (given the slow time scale dictated by $\alpha^{h}$), and inhibit the neuron when $u_{j,x}>I_{x}$ for a prolonged period. $\gamma^{h}$ is the gain of this term, controlling the strength of this homeostatic drive. We refer to this term as “h-adaptation" considering that one well-studied effect of the h-current in neurons is voltage homeostasis [20].

**Table 1:** Default model parameters.

Parameter	Value
Number of excitatory neurons, *N*_*e*_	80
Number of inhibitory neurons, *N*_*i*_	20
Excitatory rate constant, $\alpha_{e}$	100 Hz
Inhibitory rate constant, $\alpha_{i}$	200 Hz
Voltage homeostasis rate constant, $\alpha^{h}$	0.1 Hz
Spike frequency adaptation rate constant, $\alpha^{m}$	0.1 Hz
Activation function non-linear gain, β	50
Variance of noisy input, *D*	0.0001
Excitatory bias current, *I*_*e*_	-0.02
Inhibitory bias current, *I*_*i*_	1.0
Excitatory-excitatory synaptic strength, *w*_*ee*_	1.0
Excitatory-inhibitory synaptic strength, *w*_*ei*_	3.0
Inhibitory-inhibitory synaptic strength, *w*_*ii*_	-0.3
Inhibitory-excitatory synaptic strength, *w*_*ie*_	-4.7
Voltage homeostasis gain, ${\text{b}}^{\text{h}}$	-0.3
Spike-frequency adaptaiton gain, ${\text{b}}^{\text{m}}$	-0.3
Weight of voltage homeostasis, $\gamma^{h}$	1.2
Weight of spike-frequency adaptation, $\gamma^{m}$	50.0
Time step, Δt	1 ms

The variable $v_{j,x}^{m}$ is our modeled spike-frequency adaptation term, which increases whenever neuron *j* spikes by a factor defined by the gain $\gamma^{m}$. Considering that ${\text{b}}^{\text{m}}<0$ (see Table 1), this serves to inhibit the neuron whenever there has been a spike in the recent past. We refer to this term as “m-adaptation” considering that a primary effect of the m-current is imbuing neurons with spike-frequency adaptation [19].

The terms $Syn_{ex}^{j}$ and $Syn_{ix}^{j}$ represent the synaptic inputs to the cell *j* from the excitatory and inhibitory neurons, respectively. For simplicity, we implement all-to-all connectivity. The stochastic nature of this system required a high number of simulation repetitions, so we limited our spiking network to 100 neurons, with the number of excitatory neurons *N*_*e*_ = 80 and the number of inhibitory neurons *N*_*i*_=20, matching the 4:1 ratio commonly studied in E-I networks [69–71]. Accordingly, the post-synaptic inputs $Syn_{ex}^{j}$ and $Syn_{ix}^{j}$ are given by

$$Syn_{ex}^{j}=\frac{1}{N_{e}}\sum_{k=1,k\neq j}^{N_{e}}w_{ex}X_{k}(t)$$ (7)

$$Syn_{ix}^{j}=\frac{1}{N_{i}}\sum_{k=1,k\neq j}^{N_{i}}w_{ix}X_{k}(t)$$ (8)

where x=e,i dependent upon whether neuron *j* is excitatory or inhibitory. Connectivity excludes self-synapses.

The term $\chi_{u}^{j}$ represents an independent noise term sampled from *N*(0,1) for each neuron, while $\chi_{c}\in N(0,1)$ is an additional independent noise term that is identical for all neurons. The value of *c* represents the noise correlation between neuronal units. When *c* = 0, the noisy input to each neuron is determined entirely by $\chi_{u}^{j}$; conversely, when *c* = 1, and the noisy input to each neuron is identically determined by $\chi_{c}$.

For simplicity, we visualize the dynamics of the model via the population averages for the *u*_*x*_, $v_{h,x}$ and $v_{m,x}$ terms, simply calculated as $\frac{1}{N_{x}}\sum_{k=1}^{N_{x}}Y_{k}$ where *x* = *e* for the excitatory population and *x* = *i* for the inhibitory population, and *Y* is a place holder for any of the *u*_*x*_, $v_{x}^{h}$, or $v_{x}^{m}$ terms. We plot these population averages in visualizations of network dynamics, with the population averages of the *u*_*x*_ terms commonly referred to as *U*_*x*_. The *U*_*x*_ terms are also used for spectrogram analyses and analyses of the population variance (see below for details). We also refer to the total population average $U=\mathrm{.8}U_{e}+\mathrm{.2}U_{i}$ in some cases.

Parameters defining the system are found in Table 1. We note that while these parameters define what we term the “default" model, which exhibits spontaneous and irregular SLEs, this is not meant to imply this model has any special correspondence with physiological data; instead, this terminology is to facilitate the comparisons performed throughout this manuscript. All random sampling is done in Python using numpy functions [72]. Equations are integrated using the Euler-Maruyama method. In our simulations, *dt* = 0.1, scaled so that each time step Δt represents 1 ms.

### Time-frequency analyses

Our primary tool for quantitatively assessing the dynamics of the model is time-frequency analysis using the *pspectrum* function in MATLAB [73]. We perform this analysis on the mean activity of the excitatory population (*U*_*e*_), using the “spectrogram" mode with “TimeResolution" of 1.563 s and the “MinThreshold" option set to -17.5. Notable increases in power in these analyses correspond with increased spike synchrony among excitatory neurons, which we confirmed via comparison between the time-frequency analyses and spiking raster plots (an example of which is included as S2 Fig).

Thorough direct inspection of individual simulations revealed that SLEs were hallmarked by an increase in power in the 10-30 Hz band not seen during any other dynamics. This demarcates these events from brief instances of synchrony reminiscent of inter-ictal events and was consistent across our various manipulations to the model. We quantitatively identified such dynamics by: 1) Creating a time series of the summed power over this frequency range; 2) Smoothing this time series using MATLAB’s *smooth* function [73] with a “span” of 10; 3) Determining when this smoothed time series was >10. Each continuous period for which this occurred is deemed an SLE and counted for the purposes of presenting the SLE rate.

### Additional *in silico* experiments and analyses

Quantifications of network activity at “baseline”—excluding both SLEs and synchronous bursts reminiscent of inter-ictal spikes—are calculated via a “normalized standard deviation (SD)" of *U*_*e*_ and *U*_*i*_. To perform such analyses, we first generated a time-series of baseline activity via the following steps: 1) Taking the spectrogram of the mean excitatory activity as usual; 2) Eliminating SLEs, detected as outlined above; 3) Finding the maximum frequency for which the mean power over time is <0.001, which we’ll refer to as the “cutoff frequency"; 4) Creating a summed power time series from this cutoff frequency to 30 Hz (much like we did previously between 10-30 Hz for detecting SLEs); 5) Smoothing this power series as done in the detection of SLEs; 6) Excluding the time periods for which this smoothed power series is >0.01. The resulting time-series was analyzed by calculating the SD using internal MATLAB functions [73]. To facilitate comparison, these values were normalized (independently for *U*_*e*_ and *U*_*i*_) either relative to the value at *c* = 0.01 or relative to the parameter value in the “default” network.

Additional data processing was required to derive the mean trajectory of the adaptation terms around SLEs. After detecting SLEs as described above, we only considered SLEs beginning more than 50 s into the simulation to safely avoid any confounds by the the network’s initial state. We then extracted the mean activity (*U*_*e*_ and *U*_*i*_) and mean adaptation terms ($V_{e}^{h},V_{i}^{h},V_{e}^{m},V_{i}^{m}$) in three windows: the 5 s prior to detected SLE start, the duration of the SLE, and the 5 s after SLE end. The windows prior to and after SLE were directly averaged across the 57 SLEs. The time series during the SLE itself, given the variable duration of these events, were first normalized into time series of equal length (using the MATLAB [73] function *interp1* to interpolate these time series) and then these processed time series, which uniformly had 10000 time steps between the normalized “SLE Start" and “SLE End,” were averaged. Means for each value outside of SLEs (the black dotted line with black shading representing ± SD) were calculated by averaging the time-average of each time series between 50 s and simulation end (200 s) excluding SLEs. Average FRHs were created from 20 equally spaced intervals during each SLE (to account for the different duration of these events), as well as in the 5 s before and after each SLE.

### Significance testing

All tests of statistical significance were performed using the two-sample t-test via the *ttest2* function in MATLAB [73]. A standard threshold of *p* < 0.05 is used to report statistically significant differences.

## Results

### Model details

**Fig 1:**
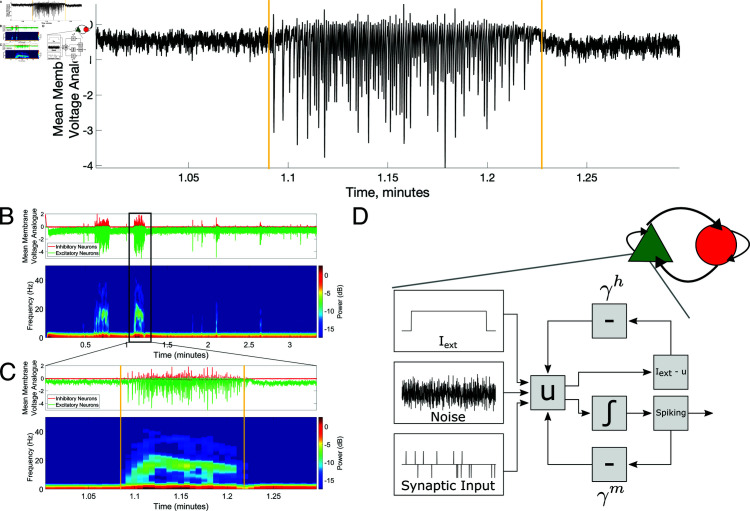
E-I microcircuit reproduces key characteristics of seizure dynamics driven by neuronal adaptation. **A**: The default model spontaneously and stochastically transitions between periods of sparse, asynchronous activity and seizure-like periods of high-frequency activity when subjected to an input with correlation *c* = .10. Mean membrane voltage analogue is the mean value of the variable *u* taken over all excitatory and inhibitory neurons and is a rough analogue of an experimental local field potential (LFP). **B**: Dynamics of the full simulation yielding Panel **A** separated between excitatory and inhibitory populations (first row) along with a spectrogram of mean activity of the excitatory population (second row). **C**: A more precise view of a single seizure-like event. Panels are as in **B**. Apparent in this visualization is a peak frequency of the seizure-like dynamics in the 10-30 Hz range and the chirp (ramp up and ramp down) of peak frequency during the seizure-like event. **D**: The model is an E-I network (diagrammed at top-right, see Materials and Methods for details) constructed of Poisson neuron models (bottom), whose activity is caricatured here via a block diagram and described in detailed equations in the Materials and Methods.

As detailed in the Materials and Methods, the model epileptogenic neuronal circuit studied here follows the canonical E-I architecture widely used to model both oscillatory and epileptiform activity [6,40,41,43,46,48,53,54]. Neuronal spiking follows a non-homogeneous Poisson process wherein the probability of spiking is dependent upon the evolution of a unitless membrane potential analogue modeled for each neuron (diagrammed in Fig 1**D**). This membrane potential analogue depends on not only a linear relaxation term, but also two forms of adaptation: spike-frequency adaptation, through which spikes in the recent past decrease the membrane potential analogue (denoted “m-adaptation” [19,35,36]); and voltage homeostasis, through which the membrane potential analogue is drawn towards the equilibrium state defined by its external driving current (denoted “h-adaptation" [20]). While a set of parameters yielding spontaneous and stochastic SLEs is highlighted in the Materials and Methods and referred to as the “default" model for convenience, we note that these parameter values were chosen not for any correspondence with physiological data (indeed, the model is dimensionless and phenomenological), but rather to facilitate comparison to a model that consistently exhibited at least one SLE per 200 s simulation.

### Excessive voltage homeostasis and spike-frequency adaptation promotes realistic *in silico* seizure-like events

In the computational study of epilepsy, particularly when using spiking neuronal microcircuits [6,52,53], *in silico* approximations of seizure commonly focus on capturing transitions in and/or out of hyperactive microcircuit oscillations and the associated increase in spike synchrony [2,41,46]. However, additional salient dynamical features of seizure dynamics differentiate these oscillations from ones serving physiological functions. One key differentiator is the non-stationary spectral characteristics of ictal activity that change over time in a stereotyped manner [74,75]. This pattern, characterized by a ramping up and slowing down of the peak frequency of ictal activity, is oftentimes termed chirps or “glissandi" [66,67]. Additionally, the most commonly classified seizure subtype, low-voltage fast-onset (LVF) seizures [74,76,77], is noted for the seeming complicity of inhibitory neurons in seizure onset [78–80] and initial peak oscillatory frequencies in the 10-30 Hz range [2,75–77,79–82].

In the default model presented here, sufficient h- and m-adaptation promotes spontaneous and irregular [64,65] transitions into SLEs exhibiting these distinguishing fluctuating spectral features of seizure. Fig 1**A** showcases one exemplar SLE generated by this model, which includes pronounced voltage homeostasis and spike-frequency adaptation via sufficient gains of the h- and m-adaptation terms; the full simulation yielding this SLE, decomposed into its excitatory and inhibitory activity, is shown in Fig 1**B**. For a majority of the 200 second simulation, activity remains asynchronous (a state we will later refer to as the model’s “baseline”), although brief instances of synchrony reminiscent of inter-ictal spikes [83] are observed. Two SLEs are observed at approximately 40 and 70 seconds, both of which terminate within 10 seconds. The frequency profiles of excitatory activity in these hyperactive states (second row, Fig 1**B**) exhibit a peak frequency just below 20 Hz and dynamics reminiscent of chirps. This is more clearly visualized by zooming in on the second SLE in Fig 1**C**. Our confidence in the classification of these events as SLEs is strengthened by the correspondence between these spectrograms and analogous experimental recordings of ictal events in human cortical [75] and subicular [84] tissue, intracerebral EEG from human hippocampus [82], and EEG from non-human primates [85,86]. This alignment with experimental data was further confirmed via quantification of the intervals between SLEs (S1 Fig), which are well fit by a gamma distribution—this matches experimental quantifications of inter-ictal distributions that also tend to be gamma distributed [87–92].

**Fig 2:**
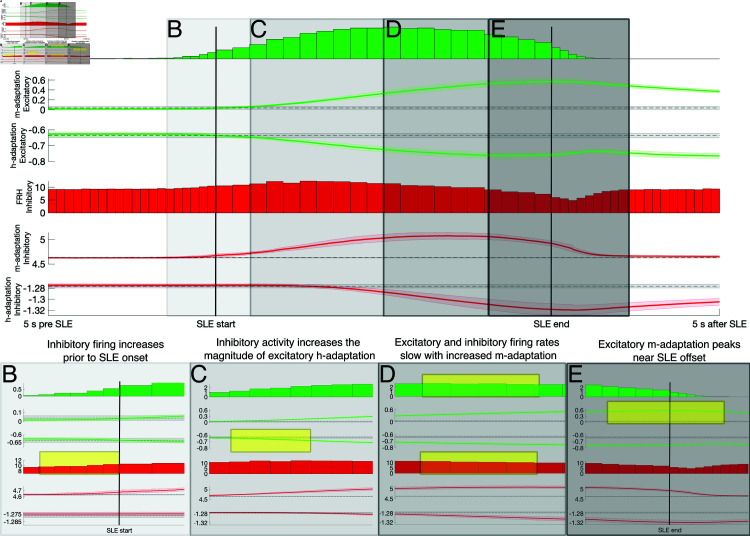
Mean firing rate activity and contribution of adaptation around SLEs. **A**: Average spiking dynamics and activity of the voltage homeostasis current (h-adaptation) and spike-frequency adaptation term (m-adaptation) around 57 spontaneously arising SLEs over 100 independent simulations with *c* = .10, separated between excitatory and inhibitory neurons. Spiking dynamics are quantified via discrete firing rate histograms (FRHs), while the population averaged activity of the h- and m-adaptation (the averaged ${\text{v}}^{\text{h}}$ and ${\text{v}}^{\text{m}}$ variables for each population, respectively) are averaged over the normalized duration of each SLE (faint shading represents ± one SD). Black dotted line (with faint shading representing ± one SD) represents the mean of these terms outside of SLEs. **B-E**: Portions of Panel **A** corresponding with four key dynamics in the evolution of the average SLE: increased inhibitory activity above the mean prior to SLE onset (manifesting in the highlighted increased inhibitory m-adaptation prior to SLE start; Panel **B**), the resulting increase in the magnitude of excitatory h-adaptation around and following SLE start (highlighted, Panel **C**), the slowing down of firing activity as m-adaptation accumulates (highlighted, Panel **D**), and the termination of the SLE associated with maximal excitatory m-adaptation (manifesting in the highlighted peak in excitatory m-adaptation around SLE end; Panel **E**).

To delineate the role of voltage homeostasis and spike frequency adaptation in these events, we captured the trajectory of the h- and m-adaptation terms during SLEs in both the excitatory and inhibitory populations. These SLEs are detected as described in the Materials and Methods as periods with sufficiently high spectral power in the 10-30 Hz band. We normalized and averaged h- and m- adaptation time series around 57 SLEs to yield a visualization of these terms’ typical progression before, during, and after an average SLE in Fig 2**A** (see details in Materials and Methods). Apparent in this visualization are important changes in h- and m-adaptation from the moments immediately preceding an SLE through its termination, in contrast with their near constant values in the 5 seconds prior to SLEs. We emphasize here that the plotted h-adaptation is the mean of the ${\text{v}}^{\text{h}}$ term for each population, and likewise the plotted m-adaptation is the mean of the ${\text{v}}^{\text{m}}$ term for each population (see details in the Materials and Methods). This quantifies the relative degree to which these adaptation terms contribute to network activity during the progression of the average SLE, but given the entirely phenomenological nature of this model these values do not have any particular physiological equivalents. These modeling choices are outlined in further detail in the Materials and Methods and expounded upon in the Discussion.

**Fig 3:**
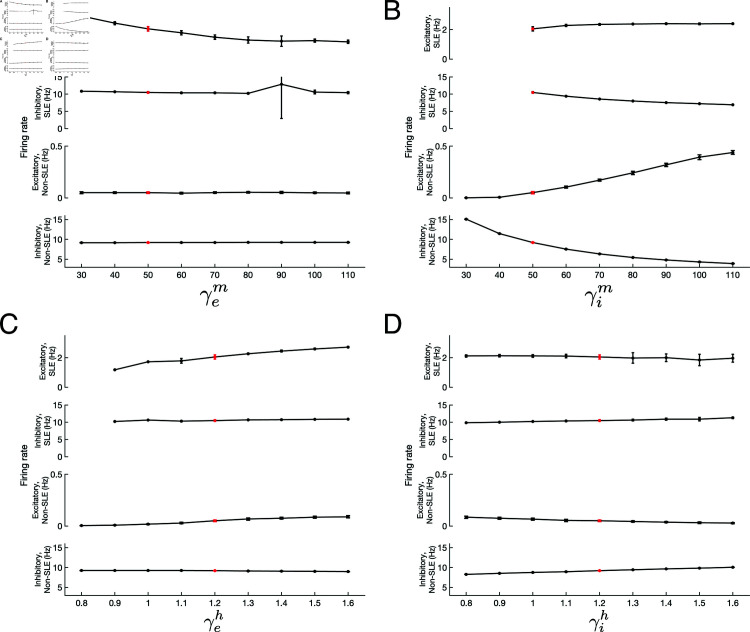
Effects of h- and m-adaptation on firing rates and excitability of excitatory and inhibitory cells inside and outside of SLEs. **A-D**: Modulation of excitatory firing rate during SLEs (first row), inhibitory firing rate during SLEs (second row), excitatory firing rate outside of SLEs (third row), and inhibitory firing rate outside of SLEs (bottom row) as the four adaptation terms ($\gamma_{e}^{m},\gamma_{i}^{m},\gamma_{e}^{h},\gamma_{i}^{h}$) are varied independently. The red point represents the default model parameters (note that the varied y-axes to explain the different error bars in each panel). Firing rates of 0 during SLEs (in Panels **B** and **C**) are indicative of no SLEs occurring for that parameter value. Plots show mean ± one SD over 25 independent 200 s simulations with *c* = .99. Changes in $\gamma_{i}^{m}$ (Panel **B**) and $\gamma_{e}^{h}$ (Panel **C**) have the most pronounced effect on network dynamics, particularly via a monotonic increase in non-SLE excitatory firing rate as these weights are increased. The most pronounced effect of $\gamma_{e}^{m}$ (Panel **A**) is a decrease in excitatory firing rate during SLEs, while the most pronounced effect of $\gamma_{i}^{h}$ (Panel **D**) is increased inhibitory firing rates throughout the simulation.

Notable in these trajectories are the following key features: 1) The first manifestation of altered activity in adaptation terms around SLE start is increased inhibitory m-adaptation (which tracks spiking activity with a time delay, as detailed in the Materials and Methods; Fig 2**B**); 2) This increased inhibitory activity promotes a build-up of the excitatory h-adaptation (which tracks voltage with a time delay; see Materials and Methods for further details) and in turn a net increase in the excitability of excitatory neurons in the moments around SLE onset (Fig 2**C**); 3) While initially inhibitory cells are unable to compensate for increased excitatory excitability due to rapid increases in the m-adaptation, the accumulation of m-adaptation eventually leads firing rates to slow (Fig 2**D**); and 4) SLE end corresponds with a peak in the excitatory m-adaptation, indicating that excitatory spike-frequency adaptation influences SLE termination (Fig 2**E**). It is also worth emphasizing that the interactions between the adaptation terms can also be analyzed to explain the distinctly seizure-like non-stationary spectral characteristics of our SLEs: the excitatory firing rate increases in the early stages of the SLE when increases in the magnitude of excitatory h-adaptation outpace those in excitatory m-adaptation, while the plateau and subsequent decrease in excitatory firing rate corresponds with a period in which the magnitude of the h-adaptation plateaus and decays while that of the m-adaptation continues to increase.

This analysis showcases a potentially complicit role of h- and m-adaptation, with cell-type specific effects, in the onset of SLEs. Given this, we would also expect these terms to have some influence on network dynamics more generally, which we assessed via network activity as a function of the gains dictating the strength of each adaptation term independently in the excitatory ($\gamma_{e}^{m},\gamma_{e}^{h}$) and inhibitory ($\gamma_{i}^{m},\gamma_{i}^{h}$) populations. As presented in the Materials and Methods, these terms represent the “weight" given to the h-/m-adaptation (superscript) in the excitatory/inhibitory population (subscript), with the magnitude of these raw values a function of the underlying mathematical implementation of these adaptive mechanisms and not indicative of their comparative strength. An increase in a particular γ increases the influence of the corresponding adaptation term, and can therefore roughly be conceptualized as an increase in the maximal conductance of the associated ionic current driving that adaptation (relative, of course, to the phenomenological abstractions of such currents necessary for this model, as presented in the Materials and Methods and examined further in the Discussion).

These analyses, presented in Fig 3, serve as evidence for the relative importance of inhibitory m-adaptation ($\gamma_{i}^{m}$) and excitatory h-adaptation ($\gamma_{e}^{h}$) in modulating network activity, both during SLE and non-SLE periods. Indeed, increases to $\gamma_{i}^{m}$ (Fig 3**B**) cause notable suppression of the inhibitory firing rate both inside and outside of SLEs, and corresponding amplification of the excitatory firing rate. Similarly, increases in $\gamma_{e}^{h}$ (Fig 3**C**) contribute to notable increases in excitatory activity both inside and outside of SLEs, although with limited effect on inhibitory firing. In comparison, the effects of changing $\gamma_{e}^{m}$ (Fig 3**A**) and $\gamma_{i}^{h}$ (Fig 3**D**) are relatively subtle, with the most apparent being a decrease in excitatory cell activity during SLEs as $\gamma_{e}^{m}$ increases and an increase in non-SLE inhibitory activity as $\gamma_{i}^{h}$ increases.

While these changes, particularly those brought about via $\gamma_{i}^{m}$ and $\gamma_{e}^{h}$, might be contextualized as increasing the system’s E-I balance and in turn it’s susceptibility to SLEs, it is noteworthy that inhibitory activity consistently dominates excitatory activity outside of SLEs: inhibitory cells fire on the order of approximately 10 Hz, while excitatory cells fire at most at 0.1 Hz outside of SLEs in each of these scenarios. Even within an SLE, excitatory cells fire consistently at approximately 2 Hz, far below the excitatory population rhythm distinguishing SLEs in the 10-30 Hz range. Furthermore, with the notable exception of $\gamma_{i}^{m}$, the effect of these gains on non-SLE activity is largely negligible. Therefore, if the gains of these adaptation terms (our phenomenological approximation of the misexpression of the h- and m-channels driving such adaptation) notably influence the system’s susceptibility to SLEs, it follows that their most salient effects are realized not at the level of single-neuron excitability or activity, but instead in network-level effects driving population activity.

### Cell-type specific overexpression of h- and m-adaptation increases SLE susceptibility

**Fig 4:**
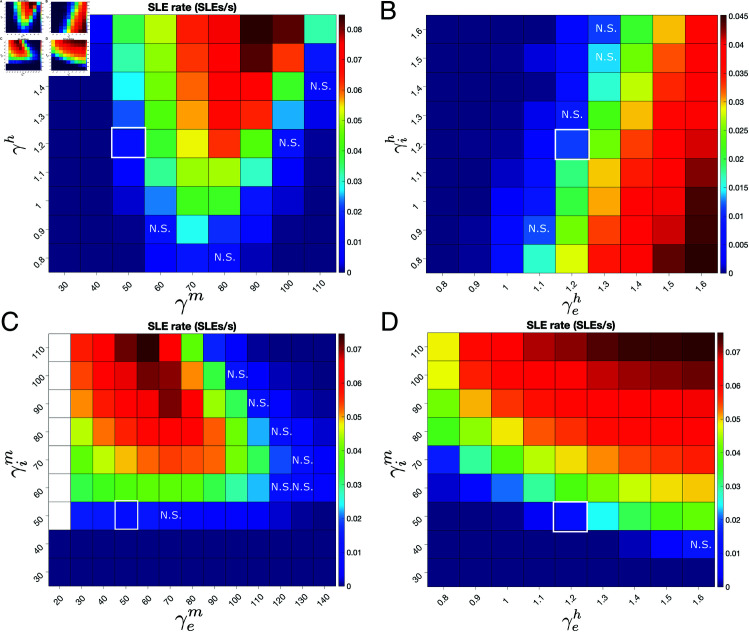
Effects of cell-type specific h- and m-adaptation gains on SLE rates. **A**: Heatmap of SLE rate (SLEs/s) as a function of the h-adaptation gain ($\gamma^{h}$) and m-adaptation gain ($\gamma^{m}$) varied uniformly for the excitatory and inhibitory populations. SLE rate is calculated over a 200 second simulation and averaged over 25 independent trials, with a high input correlation of *c* = .99 to maximize the system’s vulnerability to SLEs (as further described below). Default values ($\gamma^{h}=1.2$, $\gamma^{m}=50$; demarcated by white border) result in an average of approximately two SLEs per 200 second simulation. Increasing $\gamma^{h}$ increases SLE rate, while changes to $\gamma^{m}$ have a more complex effect. With few exceptions, changes in $\gamma^{m}$ and $\gamma^{h}$ exhibit a significantly different SLE rate from the default values (two-sample t-test, *p* < 0.05). **B**: Heatmap of SLE rate as a function of the h-adaptation gain in the inhibitory ($\gamma_{i}^{h}$) and excitatory ($\gamma_{e}^{h}$) populations. Results as in Panel **A**. Increasing $\gamma_{e}^{h}$ increases SLE rate, while increasing $\gamma_{i}^{h}$ decreases it. Default values demarcated by white border. With few exceptions changes in $\gamma_{e}^{h}$ and $\gamma_{i}^{h}$ exhibit a significantly different SLE rate from the default values. **C**: Heatmap of SLE rate as a function of the m-adaptation gain in the inhibitory ($\gamma_{i}^{m}$) and excitatory ($\gamma_{e}^{m}$) populations. Results as in Panel **A**. Increasing $\gamma_{e}^{m}$ decreases SLE rate, while increasing $\gamma_{i}^{m}$ increases it. White squares indicate anomalous networks which do not exhibit termination of seizure-like activity. Default values demarcated by white border. With few exceptions, changes in $\gamma_{e}^{m}$ and $\gamma_{i}^{m}$ exhibit a significantly different SLE rate from the default values. **D**: Heatmap of SLE rate as a function of the m-adaptation gain in the inhibitory population ($\gamma_{i}^{m}$) and the h-adaptation gain in the excitatory population ($\gamma_{e}^{h}$). Results as in Panel **A**. Increases in both gains monotonically increase the SLE rate throughout the entire parameter space, supporting the hypothesized key role for these terms in SLE onset. With one exception, changes in $\gamma_{e}^{m}$ and $\gamma_{i}^{m}$ exhibit a significantly different SLE rate from the default values.

The activity of the h- and m-adaptation before and during SLEs is preliminary evidence that this adaptation is not only necessary to capture key nuances of seizure dynamics, but potentially serves a role in the initiation of these events. This hypothesis was validated by explicitly quantifying the SLE rate (SLEs per second as calculated over a 200 s simulation) as a function of these gain terms. We first varied the gain of the h- and m-adaptations uniformly for the excitatory and inhibitory populations (Fig 4**A**), revealing that SLE rate is non-linearly dependent on h- and m-adaptation gain: the effect of $\gamma^{m}$ on SLE rate is non-monotonic, while the effect of $\gamma^{h}$ varies largely depending on $\gamma^{m}$.

These results motivate the need to disambiguate the effects of these adaptation terms in each neuron population independently. The gain of the h-adaptation in the excitatory and inhibitory populations was hence independently varied in Fig 4**B**. These analyses reveal that excitatory and inhibitory h-adaptation gains have an opposite impact on SLE rate: while increasing $\gamma_{e}^{h}$ leads to an increased SLE rate, increasing $\gamma_{i}^{h}$ has the opposite effect. This follows from the intuition derived from our analyses of Figs 2 and 3: increased $\gamma_{e}^{h}$ enhances the post-inhibitory hyperexcitability of the excitatory population as a result of hyperpolarization resulting from a period of enhanced inhibitory activity. The monotonic increase in SLE rate as a function of $\gamma^{h}$ in Fig 4**A**, despite the dichotomous effects of $\gamma_{e}^{h}$ and $\gamma_{i}^{h}$ in Fig 4**B**, indicates that the seizure-promoting effect of the h-adaptation in excitatory cells outweighs a reverse effect in inhibitory cells, and further conforms with the analyses of Figs 2 and 3.

Similarly, the effects of the m-adaptation gain are cell-type dependent. Fig 4**C** shows that increases to $\gamma_{e}^{m}$ generally reduce the SLE rate (albeit non-monotonically), while the SLE rate increases monotonically with increasing $\gamma_{i}^{m}$. This analysis explains the non-monotonic effect of varying $\gamma^{m}$ seen in Fig 4**A**: at low $\gamma^{m}$ increased inhibitory activity suppresses SLEs, while at high $\gamma^{m}$ decreased excitatory activity suppresses SLEs despite an analogous decrease in inhibitory activity. These results also conform with analysis of Figs 2 and 3 highlighting the relative importance of $\gamma_{i}^{m}$ over $\gamma_{e}^{m}$ in network activity and SLE susceptibility.

A uniform pattern emerges when we jointly vary the strength of the adaptation terms hypothesized to most directly affect SLE rate: $\gamma_{e}^{h}$ and $\gamma_{i}^{m}$. Fig 4**D** shows that increases in both these terms monotonically increase the SLE rate, conforming with the hypothesized culpability of excitatory h-adaptation and inhibitory m-adaptation in SLE onset derived from analyzing Figs 2 and 3. Collectively, these results reveal that the ictogenic potential of h and m-adaptation overexpression is directly linked to cell type. This is one potential justification for the experimental paradox that both over- and under-expression of these channels can be ictogenic: the results presented in Fig 4 do highlight how decreases in $\gamma_{i}^{h}$ and $\gamma_{e}^{m}$ can also be ictogenic. While direct experimental confirmation of this prediction has yet to be presented (likely due to the inherently general nature of commonly used genetic and pharmacological manipulations of these ion channels), recent studies have highlighted differences in HCN1 expression in excitatory pyramidal cells and inhibitory interneurons that could potentially underlie cell-type specific effects of h-channel misexpression [93]. Another possibility, detailed below, is that while channel under-expression increases the E-I balance, channel over-expression promotes seizure via an alternative pathway.

### H- and m-channelopathies promote SLE susceptibility via impaired processing of correlated input

**Fig 5:**
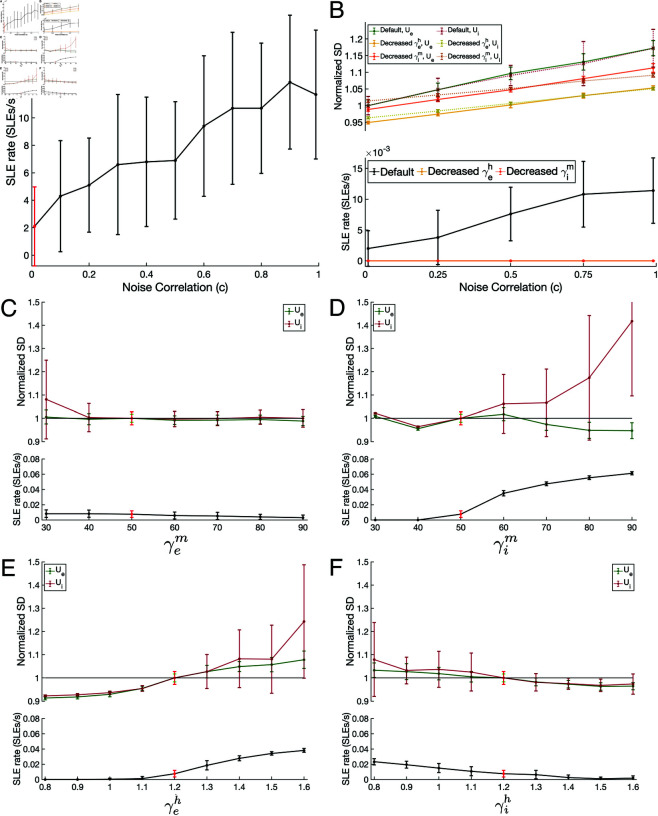
Ictogenic changes in input correlation and adaptation gains are associated with increased dynamical variability during baseline activity. **A**: SLE rate (SLEs/s) as a function of input correlation *c*. Plotted values are mean ± SD over 50 independent 200 s simulations. SLEs are significantly more frequent for each displayed *c* value in comparison to the red *c* = 0.01 case (*p* < 0.05, two sample t-test). **B**: *U*_*e*_ and *U*_*i*_ Normalized SD during baseline activity (top) and SLE rate (bottom) in the default model compared to two non-ictogenic scenarios—$\gamma_{i}^{m}$ decreased from 50.0 to 40.0 and $\gamma_{e}^{h}$ decreased from 1.2 to .8. Normalization is relative to values in the default model at *c* = .01. While the Normalized SD of both *U*_*e*_ and *U*_*i*_ behave similarly in the default network whose SLE rate increases with *c*, under non-ictogenic conditions both these measures are notably diminished. However, the effects are most pronounced in the *U*_*i*_ Normalized SD, whose gain in response to varying *c* is notably lower than for the *U*_*e*_ Normalized SD. Plots are mean ± one SD over 25 independent 200 s simulations. **C-F**: *U*_*e*_ and *U*_*i*_ Normalized SD during baseline activity (top) and SLE rate (bottom) for varying $\gamma_{e}^{m}$ (Panel **C**), $\gamma_{i}^{m}$ (Panel **D**), $\gamma_{e}^{h}$ (Panel **E**), and $\gamma_{i}^{h}$ (Panel **F**). Normalization is relative to the value of these parameters in the “default” model (bright green, *U*_*e*_ Variance; bright red, *U*_*i*_ Variance and SLE rate). Plots are mean ± one SD over 25 independent 200 s simulations with *c* = .5. Ictogenic changes in each adaptation gain uniformly cause notable increases in the *U*_*i*_ SD relative to the default model, while the *U*_*e*_ Normalized SD tracks SLE susceptibility accurately for varying $\gamma_{e}^{h}$ and $\gamma_{i}^{h}$.

With the cell type-specific effects of h- and m-adaptation gain on SLE rate established, we leveraged the *in silico* setting to derive a mechanistic explanation for the ictogenic effects of h- and m-channel overexpression. Specifically, we asked how these ictogenic changes manifest in non-SLE activity and whether such changes correspond with the mechanism for SLE onset proposed above. We first interrogated the model using a noisy input with various correlation levels, reflective of a host of potential naturally arising fluctuations in activity external to this model microcircuit [56,57,60–63] and known to induce oscillatory synchrony [59]. We found this correlation to significantly affect the SLE rate, as quantified in Fig 5**A**. With default values of each adaptation gain, all tested values of *c*>0.1 yield significant increases in the SLE rate compared to the minimally correlated case of *c* = 0.01, with any increase in *c* of at least 0.3 also yielding a statistically significant increase in the SLE rate (*p* < 0.05, two sample t-test).

We hypothesized that these ictogenic consequences were driven by pathological variability in the excitatory and/or inhibitory population response to correlated input. We quantified this variability by computing the SD of the mean excitatory and inhibitory membrane voltage analogue (*U*_*e*_ and *U*_*i*_) as a function of input correlation exclusively during “baseline” activity excluding SLEs and brief synchronous bursts reminiscent of inter-ictal events. We normalized these values independently for *U*_*e*_ and *U*_*i*_ to best visualize the relative change in these dimensionless quantities. As illustrated in Fig 5**B**, the Normalized SD of both *U*_*e*_ and *U*_*i*_ increase similarly while larger values of *c* promote higher SLE rates in the default network. Notably, changes to $\gamma_{e}^{h}$ and $\gamma_{i}^{m}$ yielding lower SLE rates coincide with the suppression of *U*_*e*_ and *U*_*i*_ variability. However, the decrease in the gain of this relationship is more notable in the *U*_*i*_ Normalized SD: this implies that a hallmarking characteristic of model systems resilient to SLEs is minimal change in the variability of inhibitory activity as a function of input correlation. The more pronounced effects of anti-ictogenic changes to the system on inhibitory activity represents additional support for the necessary role played by inhibitory signaling in our proposed SLE onset mechanism.

We next asked whether this quantification might reflect changes in network activity outside of SLEs explaining varying levels of SLE susceptibility. Choosing an intermediate *c* = .5, we systemically varied each of the four adaptation gains and measured the *U*_*e*_ and *U*_*i*_ variability alongside SLE rate (Fig 5**C-F**). As expected from the analyses presented in Fig 4, increases in $\gamma_{e}^{h}$ (Fig 5**E**) and $\gamma_{i}^{m}$ (Fig 5**D**)have the most pronounced effect on SLE rate. In each case, increased SLE rate relative to the default system is associated with a notable increase in *U*_*i*_ variability relative to the default system, and vice-versa. While more subtle, this relationship is also apparent in response to changes to $\gamma_{e}^{m}$ (Fig 5**C**) and $\gamma_{i}^{h}$ (Fig 5**E**).

When interpreted relative to the findings of Fig 5**B**, this supports the conclusion that changes in h- and m-adaptation increasing SLE susceptibility manifest in how the inhibitory population responds to correlated input. Changes to adaptation gains that increase SLE rate lead the inhibitory population to respond to correlated input with more variable activity. These variations are precisely what we propose are necessary for SLE initiation from our analysis of Fig 2. In contrast, changes to adaptation gains that decrease SLE rate lead to more regular inhibitory activity as quantified by a *U*_*i*_ Normalized SD <1, reflecting a more physiologically-relevant processing of correlated input in a manner that does not disproportionately bias network dynamics.

While the dynamics of the inhibitory population are most relevant from a mechanistic standpoint, there are obvious challenges to measuring the isolated activity of an inhibitory network in *in vivo* or *in vitro* settings. Therefore, we further analyzed the *U*_*e*_ Normalized SD as a potentially more relevant experimental biomarker of SLE susceptibility. This measure tracks SLE rate similarly to the *U*_*i*_ variability for changes in $\gamma_{e}^{h}$ and $\gamma_{i}^{h}$, but shows minimal response to changes in $\gamma_{e}^{m}$ and a non-monotonic response to changes in $\gamma_{i}^{m}$. Collectively, these results suggest that variability in baseline excitatory activity might be a stronger candidate for an experimental biomarker reflecting ictogenic changes caused by h-channelopathies rather than m-channelopathies.

**Fig 6:**
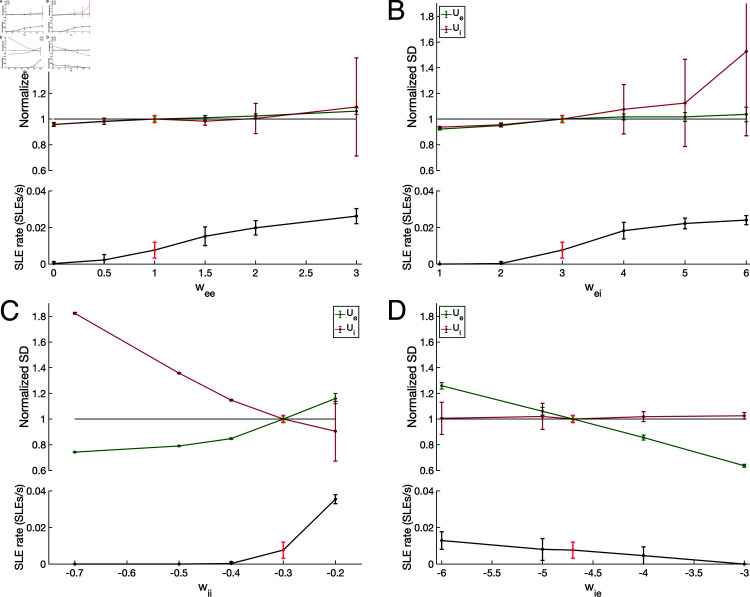
Effects of varying network topology on SLE rate and baseline population variance present additional evidence for the culpability of inhibitory activity in SLE onset. **A-D**: *U*_*e*_ and *U*_*i*_ Normalized SD during baseline activity (top) and SLE rate (bottom) for varying *w*_*ee*_ (Panel **A**), *w*_*ei*_ (Panel **B**), *w*_*ii*_ (Panel **C**), and *w*_*ie*_ (Panel **D**). Normalization is relative to the value of these parameters in the “default” model (bright green, *U*_*e*_ Variance; bright red, *U*_*i*_ Variance and SLE rate). Plots are mean ± one SD over 25 independent 200 s simulations with *c* = .5. *U*_*e*_ Normalized SD corresponds with changes to SLE rate in each of the four synaptic weights but is most pronounced for changes in inhibitory strengths (*w*_*ii*_ and *w*_*ie*_). In contrast, while *U*_*i*_ Normalized SD lacks predictive power for changes in inhibitory strengths but shows more pronounced changes in response to varied excitatory strengths (*w*_*ee*_ and *w*_*ei*_).

Finally, we subjected the default system to systematic variations in the synaptic weights (Fig 6) to determine not only whether the system’s SLE rate responds as intuited to more traditional changes in the E-I balance, but also the power of our measures of *U*_*e*_ and *U*_*i*_ baseline variability in response to other potentially ictogenic changes. While the *U*_*i*_ variability shows pronounced changes in response to ictogenic increases to the excitatory synaptic weights (*w*_*ee*_, Fig 6**A**; *w*_*ei*_, Fig 6**B**), it fails to do so in response to changes to the inhibitory synaptic weights (*w*_*ii*_, Fig 6**C**; *w*_*ie*_, Fig 6**D**). However, the *U*_*e*_ variability uniformly tracks changes to SLE rate caused by adjustments to these synaptic weights. It therefore appears that while variability in baseline inhibitory activity accurately captures dynamical changes resulting from the influence of neuronal adaptation over correlated input processing, variability in baseline excitatory activity is a more generalizable metric capturing changes in network activity associated with increased SLE rate.

The relationship between *w*_*ie*_ and SLE rate (Fig 6**D**) merits further inspection. Indeed, increases in the SLE rate as a result of an increased magnitude of *w*_*ie*_ seem in conflict with the intuitive effects one would expect based on the E-I balance. However, this phenomenon is predicted by our SLE onset mechanism, which necessarily relies upon strong inhibitory-to-excitatory signaling to create a hyperpolarized excitatory population that becomes hyperexcitable driven by the h-adaptation.

Additional analyses presented in S3 Fig indicate that inhibitory-to-excitatory signaling still plays a role in “decorrelating” input as hypothesized in physiological systems [58,94]: as *w*_*ie*_ increases, the correlation between the mean excitatory activity and the mean noisy input notably decreases, reflective of a decoupling of fluctuations in excitatory activity from fluctuations in this input. Changes to *w*_*ie*_ have by far the most pronounced effect on this relationship out of the four synaptic weights. This result conforms with our previous analyses, as one would intuitively expect more variable inhibitory activity to be required to decorrelate a correlated input which itself contains such fluctuations. We further assert that this phenomenon is fully compliant with our proposed mechanism of SLE onset in this network: while fluctuations in inhibitory activity may decorrelate excitatory activity from external input, said fluctuations can trigger the buildup of h-adaptation in the excitatory population in the process. We therefore conclude that excessive h-adaptation among excitatory cells renders the system vulnerable to a disproportionate response to the decorrelating activity of inhibitory neurons, initiating the sequence of events leading to our model SLEs. This analysis represents additional support for the hypothesis that h- and m-channel overexpression renders microcircuits vulnerable to SLEs by interfering with the proper response to a correlated input.

## Discussion

Through the presentation and analysis of a novel *in silico* model of spontaneous SLEs, this work yields new insights into the epileptogenic effects of channelopathies affecting the h- and m-channels. In this model, we find SLE onset arises when excessive h-adaptation promotes post-inhibitory hyperexcitability in excitatory neurons that cannot be compensated for by inhibitory neurons with excessive m-adaptation (Fig 2). During baseline activity, the effects of these h- and m-channelopathies manifest via the translation of correlated input into increased variability in population-averaged dynamics, which itself promotes the activity fluctuations necessary for SLE onset. This, alongside the significant effects of increased input correlation on SLE rate (Fig 5**A**), represents strong *in silico* evidence that such channelopathies promote SLEs at least in part by interfering with the typical processing of correlated input by E-I microcircuits. By articulating a pathway to SLEs driven by h- and m-channel overexpression that is independent of the changes to the E-I balance, this work represents a pivotal step towards explaining the paradoxical reality that both over- and under-expression of these channels can be ictogenic.

Indeed, while there is ample experimental evidence that over-expression of the h- and m-channels (individually) can be epileptogenic [29–33,37], these channelopathies are relatively understudied compared to channel underexpression [10,25–30,33]. In this model, independent increases to either $\gamma^{h}$ and $\gamma^{m}$ from default values (Fig 4**A**) increase SLE rate, mirroring experimental findings. The *in silico* setting allows us to further decipher the specific contributions of each adaptation term to the trajectory of SLEs. Notably, SLE-promoting increases to $\gamma_{e}^{h}$ and $\gamma_{i}^{m}$ both yield more variable inhibitory activity outside of SLEs (Fig 5**D**–**E**), a viable explanation for these ictogenic effects shared by two distinct manifestations of excessive excitability control. Further analysis yields new, computationally-supported hypotheses regarding how h- and m-channel overexpression might destructively interfere to promote seizure, with specific experimentally-testable predictions.

More generally, these findings are of crucial importance to the ongoing challenge of developing treatments for medically refractory epilepsy. Despite decades of drug development, a vast majority of anti-seizure medications remain focused on increasing GABAergic or decreasing glutamatergic signaling, with such drugs only effective for approximately 2 in 3 patients with epilepsy [38]. Such treatments are unlikely to be efficacious when the mechanism underlying seizure onset is not dependent upon an increase to the E-I balance, which is the case here: in our model, SLEs arise not from increased excitation or decreased inhibition but instead from how overexpression of the h- and m-channels alter microcircuit processing of correlated input. Delineating such mechanisms *in silico* represents a critical preliminary step towards designing clinical interventions targeted to a specific seizure impetus (i.e., h- or m-channel overexpression), an approach that could potentially lower the number of epilepsy cases deemed medically refractory.

Notably, the seizure onset mechanism outlined in this work parallels experimental findings that activation of parvalbumin positive (PV) interneurons at the seizure focus is seizure and synchrony-promoting via postinhibitory rebound [95]. In this context, this work presents the homeostatic effects of the h-current [20] as a viable impetus for this postinhibitory rebound and subsequent ictogenesis, complementing the authors’ previous presentation of a “GABAergic initiation hypothesis" for seizure [13]. How this interacts with effects of the h-channel on input resistance (h-channel underexpression is thought to render neurons hyperexcitable by increasing the input resistance) is outside the realm of this study given our idealized neuron model; however, given our group’s previous focus on capturing the dynamics of the human h-channel [96], study of these interactions remains a ripe topic for future research. Similarly, this model is able to explain the less intuitive ictogenic consequences of m-channel overexpression [37] via its cell-type specific effects: excessive spike-frequency adaptation in inhibitory cells impairs their ability to restrain excessive excitatory activity, often outweighing the effects of such adaptation in the excitatory cells themselves. While underexpression of the m-current is intuitively related to ictogenesis via spike-frequency adaptation modulated effects on neuronal excitability and has motivated the upregulation of m-channels as a target for epilepsy treatment [26,97–99], our results indicate that such interventions should ideally be targeted to excitatory neurons.

The unique role of inhibitory signaling in driving SLEs in this model (Fig 6**D**) is particularly salient given the lack of a clear consensus in the epilepsy literature as to the role of inhibitory neurons in seizure. While many studies have identified interneuronal hyperactivity prior to seizure onset and even implicated interneurons as serving a causal role in seizure initiation [11,14,68,74,79,95,100–102], others contextualize inhibitory activity as primarily “restraining" seizure [66,103] or focus on GABAergic signaling in the context of its tendency to become depolarizing during seizure propagation [104–107]. Both these phenomena have strong experimental support, and compromised inhibition undoubtedly can be epileptogenic [103,108]; however, the apparent cap in the efficacy of pharmaceutical interventions directly affecting the E-I balance [38,39] implies that the role of inhibitory neurons in seizure is likely more nuanced and complex. Our model is strong computational evidence that SLEs with biophysically realistic features can arise with intact, completely inhibitory signaling that is at least complicit—and likely causal—in SLE initiation.

Initially, this relationship between inhibitory signaling, SLE rate, and increases in baseline activity variability may seem counterintuitive. Indeed, activity in the cortex is typically uncorrelated [94] with inhibitory populations responsible for any necessary “decorrelation" [58,109,110]. In our system, while inhibitory activity still appears to play a role in decoupling excitatory activity from the noisy input (S3 Fig), it also promotes the increased variability in baseline population activity (Fig 6**D**) associated with increased SLE susceptibility. Collectively this implies that, in brain regions affected by epilepsy, seizure onset might not exclusively be driven by the failure of inhibition to decorrelate an upstream correlated input; in fact, a more nuanced corruption of this processing may play a causal role in seizure dynamics. This computationally-supported pathway merits rigorous interdisciplinary study and has the potential to recontextualize our understanding of the role of inhibition in seizure.

These conclusions are critically supported by this model’s capture of distinguishing features of seizure activity directly attributable to excessive h- and m-adaptation. Perhaps most notable among these features is the irregular nature of the SLEs, an analogue for the ongoing challenge of “seizure prediction" clinically [64,65]. Longer simulations of our model reveal that the non-periodic intervals between SLEs (an *in silico* analogue for inter-ictal intervals) are well-fit by a gamma distribution (S1 Fig), corresponding with experimental and clinical findings [87–92]. In this vein, it is worth noting that while this model exhibits higher SLE rates than one would expect *in vitro* or *in vivo*, this is purposeful to facilitate efficient *in silico* experiments given computational limitations. The model SLEs exhibit chirps [66,67] and initial oscillatory frequencies in the 10-30 Hz range [2,67,75–77,79–81,111], yielding a spectrogram in remarkable correspondence with experimental seizure recordings [75,82,84–86]. Furthermore, examination of the spiking activity of individual neuronal units in the model, particularly during approximations of inter-ictal events, reveal that different excitatory neurons participate in each event and that inhibition plays a major role in their development (S2 Fig), matching experimental findings [100,112]. Finally, the dynamics underlying the termination of our model SLEs—specifically, that the amplitude and frequency of the oscillation is non-zero at the transition out of the SLE—mirrors “Fold Limit Cycle (FLC)" seizure offset as described by [113], the most common type of seizure offset identified in their recordings. Collectively, these findings indicate that this model not only is of critical use in the study of h- and m-channelopathies in epilepsy, but also in itself represents a crucial step forward in the fundamental endeavor of capturing the features differentiating seizure from other forms of oscillatory activity in *in silico* spiking neuronal microcircuits.

This model is also particularly generalizable to a range of ictogenic insults beyond h- and m-channel overexpression, an important step towards computational models that reflect the diverse etiologies of epilepsy [3–14]. The model’s SLE rate can be affected solely by the correlation of a noisy input [109,110] and independent of changes to its amplitude, a factor controlling SLE susceptibility notably independent from the E-I balance. However, the model still responds as expected to many changes to the E-I balance [4,5] caused by varying network topology. The ability to precisely and simultaneously study multiple, and potentially interacting, pro- and anti-ictogenic effects on distinct neuronal populations has the potential to motivate a host of potentially impactful follow-up studies utilizing this model.

We note that the conclusions presented in this paper are conscientiously limited to seizure onset rather than seizure propagation. This choice justifies the use of a small microcircuit comprised of 100 neurons and the omission of the effects of changes in the chloride reversal potential [104–106,114], which are most pronounced during seizure propagation [14,115]. Coupling multiple versions of this model together to study seizure propagation [116,117] and adding the associated changes to inhibitory signaling would be an interesting follow-up to this work. While such studies would ideally be performed with a reduced version of the model, the dynamical systems techniques used to do so in our previous work [6,53] are not appropriate here given the sparse firing of the excitatory neurons outside of SLEs and the exaggerated stochasticity of the system’s dynamics. While the similarities (i.e., relative weighing of synaptic strengths) between this new model and those shown to exhibit multi-stability in our previous work [6,53] are an indicator that multi-stability underlies the transition into SLEs studied here, confirmation of this structure will require new mean-field techniques.

Similarly, it is important to acknowledge that this model lies in an important middle-ground between primarily phenomenologically [41,47] and biophysically [52,118] motivated models of seizure-like dynamics, and its applications must be correspondingly limited by the modeling focuses [119]. While this microcircuit is comprised of individual spiking neurons and captures biologically-motivated stochasticity in the transitions into and out of SLEs, it also includes purposefully idealized models capturing only the adaptive effects of the h- and m-currents. These models are designed only to capture key phenomenological effects of these currents of interest relative to the ictogenic effects of h- and m-channel overexpression. While future work will be required to determine whether the mechanisms proposed here are viable in more complex, biophysically-realistic models, the choices made in this study yield dynamics that are appropriately classified as “seizure-like” while maintaining sufficient mathematical and computational tractability, and thus are well justified given this study’s primary aims.

## Supporting information

S1 Fig Intervals between SLEs are well fit by a gamma distribution. Histogram of intervals between SLEs taken from four 10,000 second simulations of the default model with *c* = .10 reveals the non-periodic nature of seizure onset. Data is well fit by a gamma distribution (red curve) with shape parameter *a* and rate parameter *b* (95% confidence interval in brackets).
(TIFF)

S2 Fig Example raster plot illustrating spiking activity of excitatory neurons. Raster plot corresponding to example simulation illustrated in Fig 1. Of particular note is the distinct populations of excitatory cells participating in “bursts” of activity reminiscent of inter-ictal spikes (between 100 and 160 s).
(TIFF)

S3 FigInhibitory synapses control decorrelation between *U*_*e*_ and mean noisy input.**A–D**: Plots of the Pearson correlation coefficient between *U*_*e*_ (during baseline activity) and the mean (over all neurons during baseline activity) noisy input to the microcircuit as a function of the input correlation *c* (x-axis) and synaptic strengths (color coding). Plots are means ± standard deviation over 25 independent 200 s simulations. Changes to *w*_*ee*_ (Panel **A**) and *w*_*ei*_ (Panel **B**) minimally affect this correlation, with perhaps a minor trend towards decorrelation for decreased synaptic weights that are anti-ictogenic. Changes to *w*_*ii*_ (Panel **C**) are more appreciable, with decreased *w*_*ii*_ (an ictogenic change) causing decorrelation. Changes to *w*_*ie*_ (Panel **D**) exert the most control over this correlation, with ictogenic increases to *w*_*ie*_ notably decorrelating *U*_*e*_ from the mean noisy input.(TIFF)
