# Supplementary figures and images for: H- and m-channel overexpression promotes seizure-like events by impairing the ability of inhibitory neurons to process correlated inputs
Source: PLoS Comput Biol. 2025 Jun 30;21(6):e1013199. doi: 10.1371/journal.pcbi.1013199 (PMC12258601; doi:10.1371/journal.pcbi.1013199)

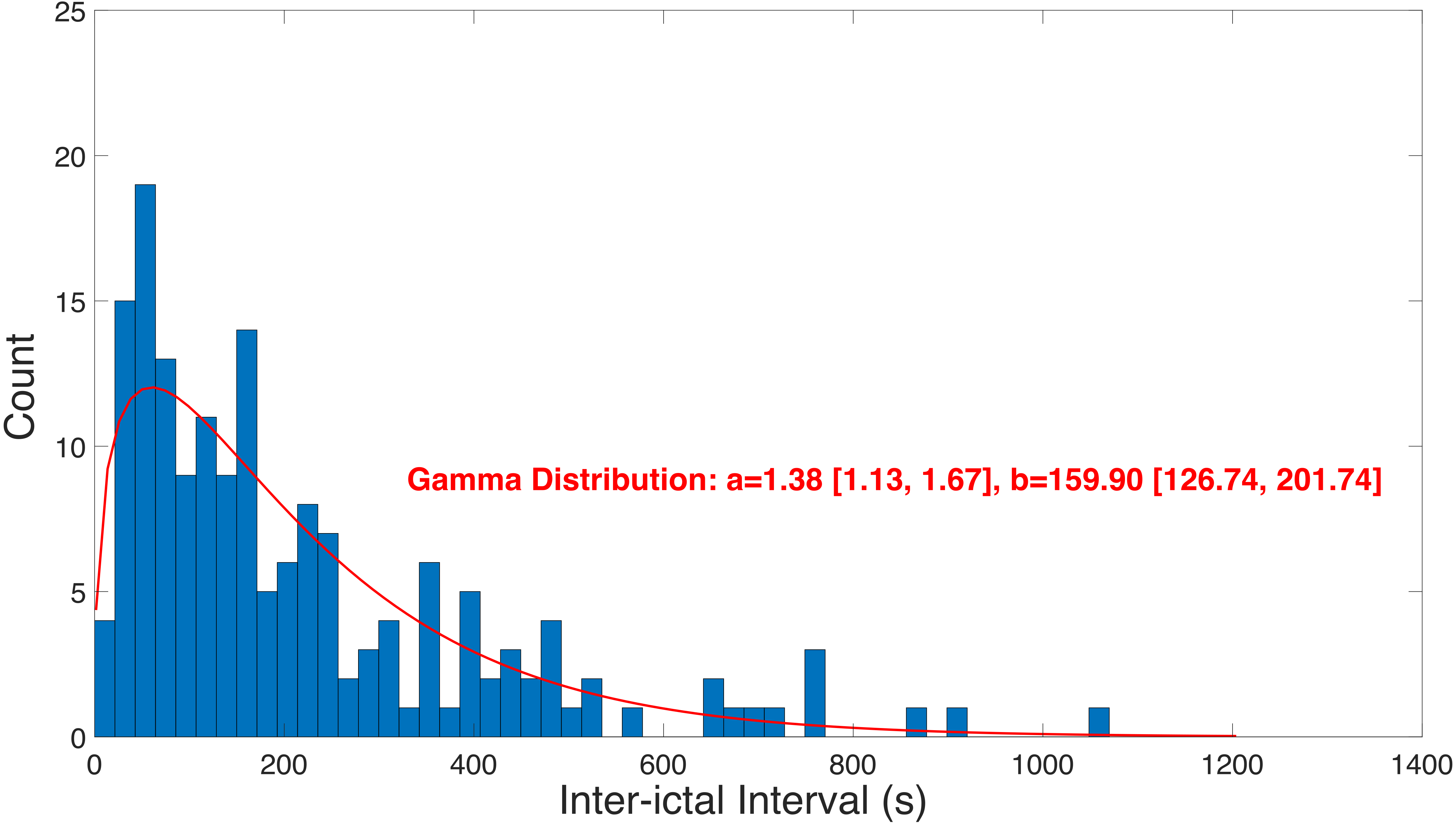

Supplement: S1 Fig — Histogram of intervals between SLEs taken from four 10,000 second simulations of the default model with c = .10 reveals the non-periodic nature of seizure onset. Data is well fit by a gamma distribution (red curve) with shape parameter a and rate parameter b (95% confidence interval in brackets). (TIFF) [file pcbi.1013199.s001.tiff]

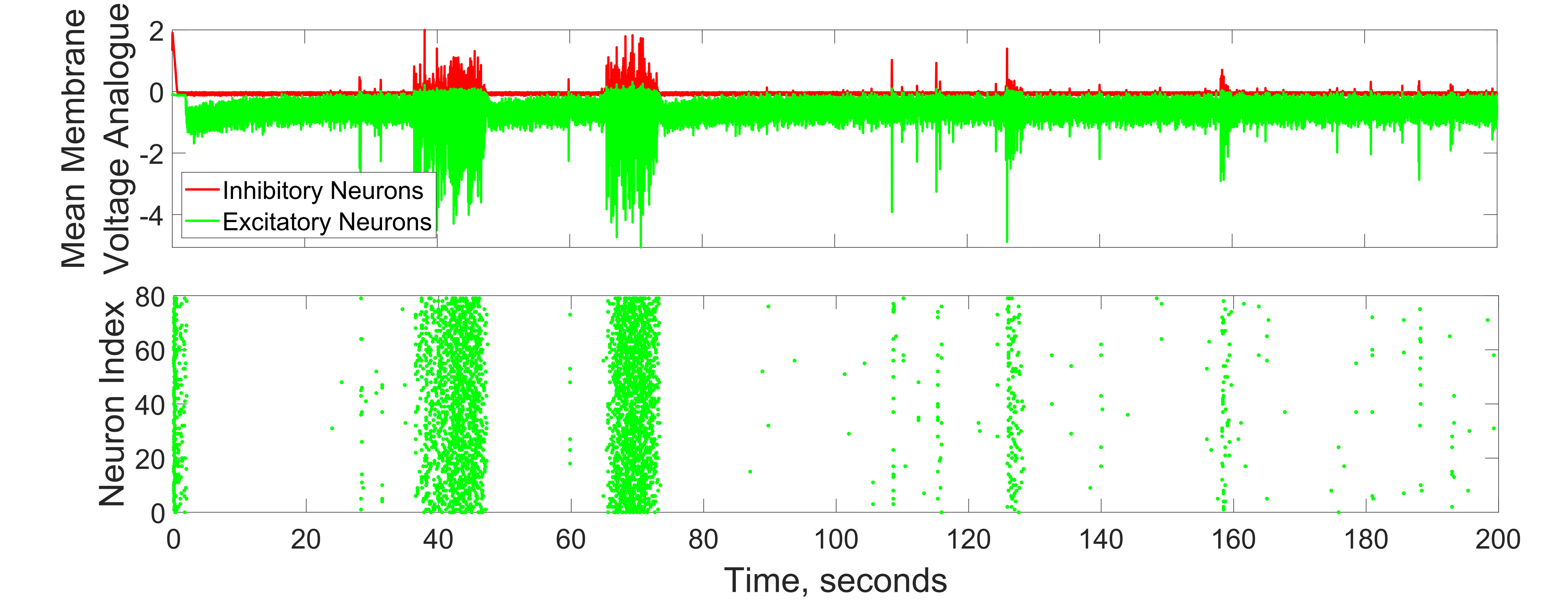

Supplement: S2 Fig — Raster plot corresponding to example simulation illustrated in Fig 1. Of particular note is the distinct populations of excitatory cells participating in “bursts” of activity reminiscent of inter-ictal spikes (between 100 and 160 s). (TIFF) [file pcbi.1013199.s002.tiff]
